# Effects of Cooking Cycle Times of Marinating Juice and Reheating on the Formation of Cholesterol Oxidation Products and Heterocyclic Amines in Marinated Pig Hock

**DOI:** 10.3390/foods9081104

**Published:** 2020-08-12

**Authors:** Xiuyun Guo, Yawei Zhang, Ye Qian, Zengqi Peng

**Affiliations:** 1 College of Food Science and Technology, Nanjing Agriculture University, Nanjing 210095, China; guoxiuyun@njau.edu.cn (X.G.); 2016108045@njau.edu.cn (Y.Q.); zqpeng@njau.edu.cn (Z.P.); 2 Synergetic Innovation Center of Food Safety and Nutrition, Nanjing 210095, China

**Keywords:** processing method, hazardous compounds, GC-MS, HPLC, marinated pig hock

## Abstract

In this work the effects of cooking cycle times of marinating juice and reheating on the formation of cholesterol oxidation products (COPs) and heterocyclic amines (HAs) in marinated pig hock were investigated. After the 12th cycle, the total content of COPs was 3.3, 2.0, and 2.0 times higher than that after the 1st cycle in the skin, subcutaneous fat, and lean meat, respectively. The total content of HAs was 5.8, 6.0, and 5.6 times higher than that after the 1st cycle in the skin, subcutaneous fat, and lean meat, respectively. Notably, more COPs were present in the lean meat than in the skin and subcutaneous fat, whereas the content of HAs in the skin was the highest. Compared with the unreheated samples, the total content of COPs and HAs in all tissues increased after reheating at 95 °C for 30 min or at 121 °C for 25 min, but no significant difference was found between different reheating conditions.

## 1. Introduction

High cholesterol oxidation products (COPs) and heterocyclic amines (HAs) intake generated from meat processing poses a risk to human health. COPs in meat products have caused some concern due to undesirable biological implications such as atherosclerosis, cancer, and neurological disorders, and some other harmful effects for human health [1,2,3]. HAs consumption is associated with the increased DNA damage [4], and with tumors of the colon, prostate, lungs, skin, breast, liver, and gastrointestinal tract [5]. It was reported that cardiovascular diseases and malignant tumors were the major causes of death in China. Data from the National Cardiovascular Prevention Center revealed that about 290 million patients suffered from cardiovascular diseases (CVD), which was still the leading cause of death in 2016, more than tumors or any other diseases. CVD-related deaths accounted for more than 40% of all deaths [6]. In addition, the burden of cancer showed a continuous upward trend in China, and about 2,338,000 cancer deaths were reported in 2015, accounting for 23.91% for all deaths [7]. Therefore, it’s meaningful to prevent the formation of COPs and HAs during meat processing. Many studies show that the contents of COPs and HAs can apparently increase after food processing [8,9]. Thermal processing always causes the oxidation of cholesterol and the formation of HAs [10,11,12], and the formation e of COPs and HAs is related to the heating parameters (temperature/time) and cooking methods [11,13,14,15].

Marinated pig hock is a traditional Chinese food commodity dating back to thousands of years ago. The traditional marinating procedure is shown as follows: the fresh pig hock is immersed in a marinade for a certain period of time, and then the pig hock is braised in the marinating juice for 1 h or longer at 90~100 °C [16]. Then, the well-done pig hock can be sold or reheating for a long shelf life, such as canned meat. Conventionally, the marinating juice is used for several or dozens of cycles, or even several years, which not only confers good flavor to marinated pig hock, but also generates COPs and heterocyclic amines HAs. In previous studies, COPs and HAs were detected in pig pork and feet heated in a marinating juice that contained soy sauce and crystal sugar, and the soy sauce and crystal sugar inhibited the formation of COPs but enhanced the formation of HAs in pig pork and feet [16,17]. Additionally, Yao et al. [18] found that the total content of HAs in braised chicken was approximately 7 times higher when its soup was boiled for 20 cycles than when it was boiled for 5 cycles. Thermal sterilization, that is, reheating, has been widely used to extend the shelf life of meat products. However, it will cause questionable overall quality. Flavour, aroma, and texture are negatively affected after this process [19]. However, few works have explored the effects of reheating on the formation of harmful substances. In our previous study, it was found that commercially braised sauce beef sterilized by heating at 121 °C had a relatively higher total HAs content than unsterilized samples [20]. Nevertheless, the effects of cooking cycle times of marinating juice and reheating, which are popular operations in Asia, on the formation of COPs and HAs in marinated pig hock, have not yet been reported. 

Therefore, the aim of this work was to investigate the effects of cooking cycle times of marinating juice and reheating on the formation of COPs and HAs in marinated pig hock skin, subcutaneous fat, and lean meat. The data of present study could be used to identify conditions that minimize COPs and HAs formation in marinated pig hock.

## 2. Materials and Methods

### 2.1. Materials and Reagents

Fresh boned pig hocks from four and a half month-old three-crossbred pigs with an average weight of 1.2 ± 0.2 kg were selected from a local slaughterhouse (Nanjing, Jiangsu, China). Soy sauce (total nitrogen 1.2 g/100 mL, ammoniacal nitrogen 0.58 g/100 mL, non-ammoniacal nitrogen 0.62 g/100 mL, total acid 2.18 g/100 mL, salt content 16.3 g/100 mL), sugar, salt, and a spice mixture (ginger, star anise, cinnamon, pepper) were obtained from a local supermarket in Nanjing. 

COPs standards, namely, 7β-OH (7β-hydroxycholestero), triol (3β,5α,6β-trihydroxycholestane), and 25-OH (25-hydroxycholesterol) were from Toronto Research Chemicals (Downsview, ON, Canada). 5,6α-EP (cholesterol-5α,6α-epoxide) was from TCI (Shanghai, China), and 7-keto (7-ketocholesterol) was from Santa Cruz Biotechnology (Santa Cruz, CA, USA). The COPs derivatization agent Sylon BTZ (BSA+TMCS+TMSI, 3:2:3 *v*/*v*/*v*) was from Supelco (Bellefonte, PA, USA). Twelve HAs standards, including IQ (2-amino-3-methylimidazo [4,5-f]quinoline), MeIQ (2-amino-3,4-dimethylimidazo[4,5-f]quinoline), MeIQx (2-amino-3,8-dimethylimidazo[4,5-f]quinoxaline), 4,8-DiMeIQx (2-amino-3,4,8-trimethylimidazo[4,5-f]quinoxaline), 7,8-DiMeIQx (2-amino-3,7,8-trimethylimidazo[4,5-f]quinoxaline), PhIP (2-amino-1-methyl-6-phenylimidazo[4,5-b]pyridine), Norharman (9H-pyrido[3,4-b]indole), Harman (1-methyl-9H-pyrido[3,4-b]indole, Trp-P-2 (3-amino-1-methyl-5H-pyrido[4,3-b]indole acetate), Trp-P-1 (3-amino-1,4-dimethyl-5H-pyrido[4,3-b]indole acetate), AαC (2-amino-9H-pyrido[2,3-b]indole) and MeAαC (2-amino-3-methyl-9H-pyrido[2,3-b]indole), were from Toronto Research Chemicals (Downsview, ON, Canada). High-performance liquid chromatography (HPLC)-grade reagents, including ethyl acetate and pyridine, were acquired from Aladdin Bio-Chem Technology Co., Ltd. (Shanghai, China). Methanol and acetic acid were purchased from Tedia Co. (Fairfield, OH, USA). Acetonitrile was obtained from ROE Co. (Newark, DE, USA). Other analytical grade chemicals (such as chloroform, n-hexane, anhydrous ether, acetone, dichloromethane, methylbenzene, ammonium acetate, sodium hydroxide, hydrochloric acid, and ammonia) were obtained from Sinopharm Chemical Reagent Co., Ltd. (Shanghai, China).

### 2.2. Sample Preparation

To study the effects of cooking cycle times of marinating juice on the formation of COPs and HAs, 36 pig hocks were prepared. In the first cycle, 5 L of fresh marinating juice was prepared by mixing 90 g of salt, 70 g of soy sauce, 30 g of sugar, and 5 g of the spice mixture in a 10-L stainless steel pot with a lid and boiling the mixture using an induction cooker. After being precooked in pure water at 100 °C for 30 min, 3 hocks were cooked in the fresh marinating juice at 98 ± 2 °C for 2 h and then at 90 °C for 30 min. Subsequently, the marinating juice was filtered and recycled used to cook 11 more groups of hocks. For each cycle, 3 precooked hocks together with 9 g of salt, 7 g of soy sauce, 3 g of sugar and 0.5 g of spice were added to the filtered marinating juice, which was supplemented with water to 5 L, and then cooked at 98 ± 2 °C for 2 h and then at 90 °C for 30 min. After cooling for 4 h to ambient temperature, the hocks were collected after the 1st, 4th, 8th, and 12th cycles for COPs and HAs analysis.

### 2.3. Reheating

To investigate the effects of reheating on the formation of COPs and HAs, 9 pig hocks were prepared. The fresh marinating juice and hocks were cooked as described above. After cooling for 4 h to ambient temperature, 6 hocks were individually vacuum-packaged and subsequently reheated for 30 min at 95 °C in a water bath (3 hocks) or 121 °C for 25 min (using an autoclave) (3 hocks), respectively. The other 3 hocks served as a control and were not reheated. The COPs and HAs were determined after the samples were stored at 4 °C for 24 h.

### 2.4. Determination of COPs

The samples were extracted and purified using the method reported by Lee et al. [13]. After purification, the extract was evaporated to dryness under nitrogen and then dissolved in 2 mL of pyridine. Finally, 50 μL of the sample was poured into a 250-μL vial insert, and 50 μL of Sylon BTZ was added. The mixture was then placed in the dark at 25 °C for 1 h to ensure complete derivatization. Afterwards, the samples were analyzed using a gas chromatography-mass spectrometry (GC-MS) chromatogram as described by Lee et al. [13]. A HP-5 MS capillary column was used to separate 5 COPs, namely, 7β-OH, 5,6α-EP, triol, 25-OH, and 7-keto. The recovery was performed by adding a mixture of COPs standards dissolved in ethyl acetate (1 mL) at three different concentrations (1000, 4000, and 20,000 ng/mL for 5,6α-EP and 350, 4000 and 20,000 ng/mL for the other COPs) to 5 g of a blank sample (not containing the 5 COPs) for extraction, purification and determination. Seven 50 μL COPs standards with different concentrations (20, 12, 4, 0.8, 0.4, 0.1 and 0.06 mg/L) were prepared to determine the standard curves. Linear regression (concentration of the compound vs. peak area of the quantitative ion) equations were calculated for the individual COPs in the mixed stock solutions. The concentrations of the COPs in the samples were calculated using the linear regression equations.

### 2.5. Determination of HAs

The extraction and purification of HAs was performed according to the method described by Yao et al. [20]. After solid-phase extraction, the samples were analyzed using high-performance liquid chromatography (HPLC). The determination and quantification were performed according to the method described by Yao et al. [20].

The recovery was performed by adding a mixture of HAs standards dissolved in methanol (0.1 mL) at three different concentrations (400, 2000, and 10,000 ng/mL for IQ, MeIQ, MeIQx, 4,8-DiMeIQx and 7,8-DiMeIQx and 40, 200 and 1000 ng/mL for Norharman, Harman, Trp-P-2, PhIP, Trp-P-1, AαC and MeAαC) to 2 g of a sample for extraction, purification and determination. Eight HAs standards with different concentrations (1000, 500, 200, 100, 50, 20, 10, and 1 ng/mL for IQ, MeIQ, MeIQx, 4,8-DiMeIQx and 7,8-DiMeIQx; 100, 50, 20, 10, 5, 2, 1, and 0.1 ng/mL for Norharman, Harman, Trp-P-1, PhIP, Trp-P-2, AαC and MeAαC) were prepared to determine the standard curves. Linear regression (concentration of the compound vs. peak area) equations were calculated for the individual HAs in the mixed stock solutions. The concentrations of the HAs in the samples were calculated using the linear regression equations.

### 2.6. Statistical Analysis

The experiments were performed in triplicate. Statistical analysis was performed using Statistical Analysis System (SAS Institute Inc., Cary, NC, USA). One-way analysis of variance (ANOVA) was carried out, and Duncan’s multiple range test was used to evaluate the differences between treatments. *p*-value < 0.05 was considered statistically significant.

## 3. Results

### 3.1. Effects of Cooking Cycle Times of Marinating Juice on the Formation of Cops in Marinated Pig Hock Skin, Subcutaneous Fat and Lean Meat

**Figure 1:**
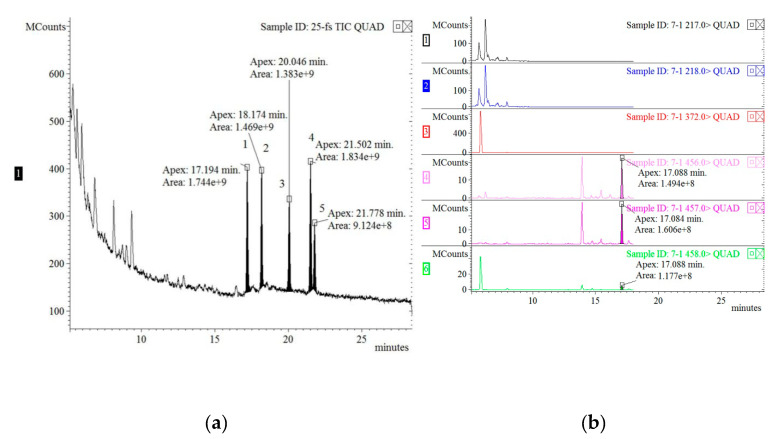

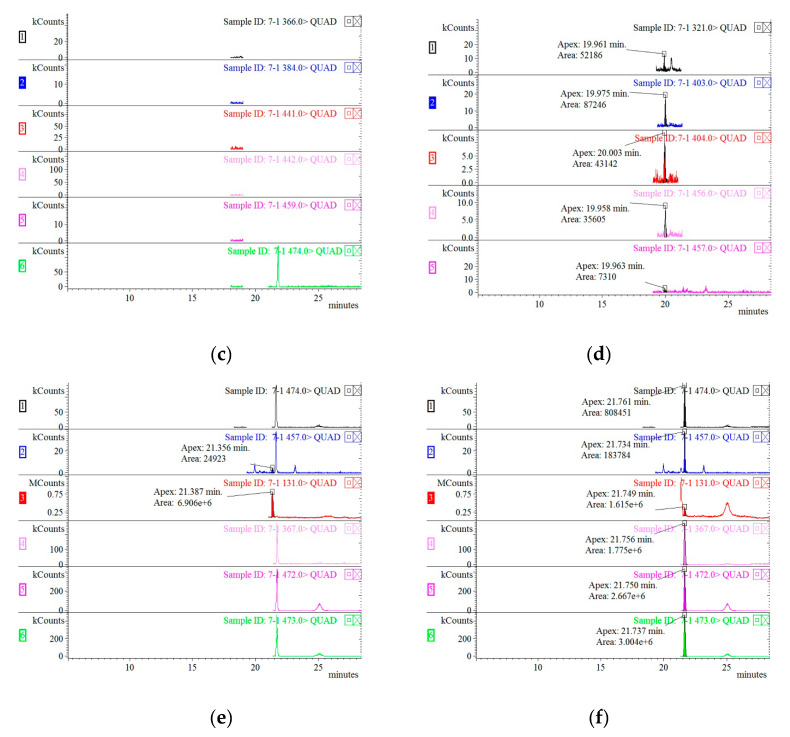
(**a**) Gas chromatography-mass spectrometry (GC-MS) chromatogram of five cholesterol oxidation products (COPs) standards detected by full-scan mode. Peaks: 1, 7β-OH; 2, 5,6α-EP; 3, triol; 4, 25-OH; and 5, 7-keto. (**b**–**f**) GC-MS chromatograms of five COPs in a hock skin after 8 th cycle detected by selected ion monitoring mode. b, 7β-OH; c, 5,6α-EP; d, triol; e, 25-OH; and f, 7-keto.

The GC-MS chromatogram of five COPs standards detected by full-scan mode and a representative sample detected by selected ion monitoring mode are shown in Figure 1. 7β-OH, 5,6α-EP, triol, 25-OH, and 7-keto were separated within 22 min with retention times of 17.194, 18.174, 20.046, 21.502, and 21.778 min, respectively (Figure 1). The correlation coefficients for all five COPs were higher than 0.99. The recoveries of the five COPs from the skin, subcutaneous fat, and lean meat were between 61.16% and 96.96%, and the relative standard deviations (RSDs) of all the samples were less than 7.80%. The limits of detection (LODs) of 7β-OH, 5,6α-EP, triol, 25-OH, and 7-keto were 0.02, 47.07, 4.19, 5.68, and 0.41 ng/g, respectively.

The effect of cooking cycle times of marinating juice on the formation of COPs is shown in Table 1. 5,6α-EP was not detected in any of the samples from the three tissues. 7β-OH, 25-OH, and 7-keto were detected in all the samples, while Triol was not formed until the 4th cycle. In general, the content of 7β-OH, 25-OH, and 7-keto in the three tissues increased with the cooking cycle times of marinating juice.

After 12 cycles, the content of 7β-OH, 25-OH, and 7-keto in the skin increased by 324.7%, 25.7%, and 227.3% (*p* < 0.05), respectively, relative to the 1st cycle. In addition, the total content of COPs reached 1968.4 ng/g, which was 3.3 times higher than that after the 1st cycle.

In the subcutaneous fat, after 12 cycles, the content of 7β-OH, 25-OH, and 7-keto increased by 96.5%, 21.5%, and 88.4%, respectively, relative to the 1st cycle. The total content of COPs after 12 cycles was 2.0 times higher than that after the 1st cycle.

In the lean meat, the content of 7β-OH, 25-OH, and 7-keto after 12 cycles increased by 93.8%, 14.1% and 80.4%, respectively, relative to the 1st cycle. The total content of COPs after 12 cycles was 2.0 times higher than that after the 1st cycle.

### 3.2. Effects of Cooking Cycle Times of Marinating Juice on the Formatmion of Has in Marinated Pig Hock Skin, Subcutaneous Fat and Lean Meat

**Figure 2:**
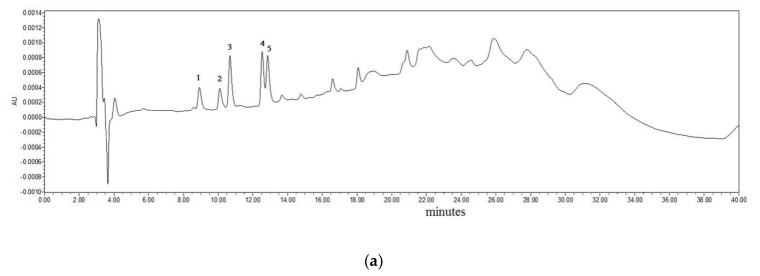

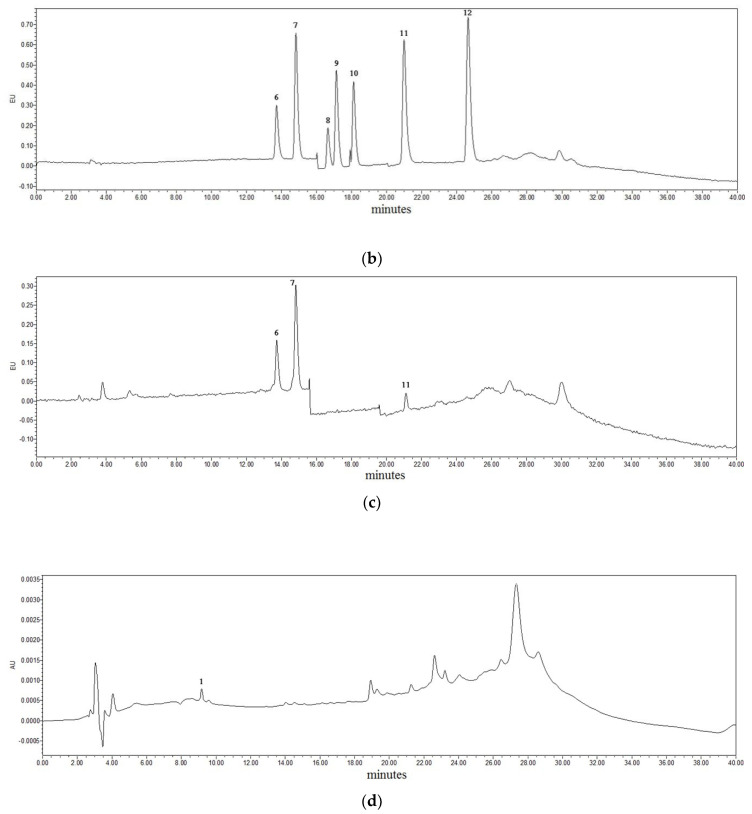
HPLC chromatogram of 12 HAs. (**a**) HAs standards detected with UV detection in 263 nm. (**b**) HAs standards detected with fluorescence detection. (**c**) HAs in a hock skin after 8th cycle detected with UV detection in 263 nm. (**d**) HAs in a hock skin after 8th cycle detected with fluorescence detection. Peaks: 1, IQ; 2, MeIQ; 3, MeIQx; 4, 7,8-DiMeIQx; 5, 4,8-DiMeIQx; 6, Norharman; 7, Harman; 8, Trp-P-2; 9, PhIP; 10, Trp-P-1; 11, AαC; 12, MeAα.

The HPLC chromatograms of a mixture of HA standards and a representative sample are shown in Figure 2. IQ, MeIQ, MeIQx, 7,8-DiMeIQx, 4,8-DiMeIQx, Norharman, Harman, Trp-P-2, PhIP, Trp-P-1, AαC and MeAαC were separated within 26 min with retention times of 8.654, 9.819, 10.462, 12.319, 12.621, 13.415, 14.507, 16.320, 16.768, 17.778, 20.486 and 24.114 min, respectively. The correlation coefficients for all 12 HAs were higher than 0.99. The recoveries of the 12 HAs from the skin, subcutaneous fat and lean meat were between 68.69% and 97.81%, and the relative standard deviations (RSDs) of all the samples were less than 7%. The limits of detection (LODs) of IQ, MeIQ, MeIQx, 7,8-DiMeIQx, 4,8-DiMeIQx, Norharman, Harman, Trp-P-2, PhIP, Trp-P-1, AαC and MeAαC were 2.47, 1.18, 1.04, 0.97, 0.85, 0.06, 0.04, 0.02, 0.04, 0.03, 0.03 and 0.01 ng/g, respectively.

The effect of cooking cycle times of marinating juice on the formation of HAs in the marinated hock skin, subcutaneous fat and lean meat was shown in Table 2. The results showed that a relative high content of HAs was detected in all the samples. Norharman, Harman and AαC were detected in all the samples while IQ was decteced until the 4th cycle. The content of IQ, Norharman and Harman in 3 tissues increased with the cooking cycle times of marinating juice while no significant difference (*p* > 0.05) was found in terms of AαC content.

After 12 cycles, the content of IQ in the skin increased (*p* < 0.05) by 69.4% relative to the 4st cycle, while the content of Norharman and Harman increased by 261.0% and 232.3%, respectively, relative to the 1st cycle. The total content of HAs reached 53.16 ng/g, which was 5.8 times higher than that after the 1st cycle.

In the subcutaneous fat, after 12 cycles, the content of IQ increased by 50.0% relative to the 4st cycle, while the content of Norharman and Harman increased by 288.3% and 237.5%, respectively, relative to the 1st cycle. The total content of HAs reached 37.23 ng/g, which was 6.0 times higher than that after the 1st cycle.

In the lean meat, after 12 cycles, the content of IQ increased by 44.1% relative to the 4st cycle, while the content of Norharman and Harman increased by 260.2% and 243.2%, respectively, relative to the 1st cycle. The total content of HAs reached 32.15 ng/g, which was 5.6 times higher than that after the 1st cycle.

### 3.3. Effects of Reheating on the Formation of Cops in Marinated Pig Hock Skin, Subcutaneous Fat and Lean Meat

The effect of reheating on the formation of COPs in the marinated pig hock skin, subcutaneous fat and lean meat was shown in Table 3. 5,6α-EP and triol were not detected in any of the samples, while 7β-OH, 25-OH and 7-keto could be detected in all samples. Overall, reheating promoted the formation of 7β-OH, 25-OH, and 7-keto.

In the skin, the content of 7β-OH, 25-OH, and 7-keto increased (*p* < 0.05) by 21.6%, 10.2%, and 38.2% after reheating at 95 °C for 30 min, and increased by 17.3%, 15.9%, and 33.6% after reheating at 121 °C for 25 min, respectively. The content of total COPs increased by 26.8% and 24.6% after reheating at 95 °C for 30 min and 121 °C for 25 min.

In the subcutaneous fat, the content of 7β-OH, 25-OH increased (*p* < 0.05) by 24.6% and 8.2% after reheating at 95 °C for 30 min, and increased by 26.0% and 4.6% after reheating at 121 °C for 25 min, respectively. For 7-keto, no significant difference was found after reheating at 95 °C for 30 min, while a remarkable increase was detected after reheating at 121 °C for 25 min. The content of total COPs increased by 10.0% and 14.1% after reheating at 95 °C for 30 min and 121 °C for 25 min respectively.

In the lean meat, the content of 7β-OH and 7-keto increased (*p* < 0.05) by 55.9% and 18.1% after reheating at 95 °C for 30 min, and increased by 89.7% and 22.0% after reheating at 121 °C for 25 min, respectively. For 25-OH, no significant difference was found after reheating. The content of total COPs increased by 32.5% and 48.5% after reheating at 95 °C for 30 min and 121 °C for 25 min respectively.

### 3.4. Effects of Reheating on the Formation of Has in Marinated Pig Hock Skin, Subcutaneous Fat and Lean Meat

The effect of reheating on the formation of HAs is shown in Table 4. A relatively high amount of HAs were detected in the reheated samples. Only Norharman, Harman, and AαC were detected in all samples, and the content of Norharman, Harman and AαC increased with increasing reheating temperature in most samples, which was in accordance with previous studies [21,22].

In the marinated hock skin, the content of Norharman, Harman, and AαC were present increased (*p* < 0.05) by 19.0%, 53.7%, and 100.0% after reheating at 95 °C for 30 min, and 56.3%, 101.3%, and 125.0% after reheating at 121 °C for 25 min, respectively. In the subcutaneous fat, the content of Norharman, Harman, and AαC increased by 18.9%, 16.0%, and 64.0% after reheating at 95 °C for 30 min, and 28.5%, 25.4%, and 68.0% after reheating at 121 °C for 25 min, respectively. In the lean meat, no significant difference (*p* > 0.05) was found in terms of Norharman and Harman content, while an increase in AαC content was shown. After reheating, the total content of HAs in all tissues was higher than in unreheated samples.

## 4. Discussion

In general, three COP were easily detected in all the samples. For the cooking cycle times of marinating juice, 7-Keto, 7β-OH, and 25-OH were detected from the 1st cycle. As the most toxic COP to endothelial cells [23], Triol was not formed until the 4th cycle, implying that recycling the marinating juice should be avoided. For reheating, only 7-Keto, 7β-OH, and 25-OH were detected in all the samples. 7-Keto was the most abundant in most samples, followed by 7β-OH, 25-OH, triol and 5,6α-EP. These results are similar to those of Lee’s study [13], which showed that 7-keto and 7β-OH were the most abundant COPs in marinated ground pork, and 5,6α-EP was not detected in a 24-h heating period. As the most representative oxysterol (>30% of total COPs) in meat products, 7-keto was easily formed and could be absorbed from dietary intake of cholesterol-rich foods [24,25]. Therefore, 7-keto was often used as a marker of the total oxidative process, and particular attention had been focused on this compound [26,27].

The results clearly showed that the cooking cycle times of marinating juice and reheating treatment increased the level of cholesterol oxidation. During the cooking process, the fat soluble substances would leach from pig hock to the marinating juice. The recycled utilization of marinating juice would make it rich in the precursors, facilitating the formation of COPs. These COPs could possibly transform to the pig hock. For reheating, Min et al. [14] found that the total content of COPs in pork loin reheated by pan roasting (170 °C for 20 min) reached 715.37 µg/100 g, which was 2.5 times higher than that in a sample that was not reheated. The study of Chen et al. [17] also supported these findings, which reported that the total content of COPs in marinated pig feet skin increased from 516.8 to 1497.8 ng/g as the heating time was increased from 0 h to 24 h, suggesting that the heating time is a significant factor affecting COP formation. In addition, it was confirmed that oxidation processes during cooking are more affected at longer times than at shorter times [28]. Reheating included heating, cooling, and reheating processes. Therefore, the heating time increased, and the pork was subjected to long-term contact with oxygen, which explained the serious oxidation of cholesterol.

Comparatively, the total content of COPs in the lean meat was the highest, while that in the subcutaneous fat was the lowest. On one hand, the difference might be due to the different free fatty acids (FFAs) composition. The content of monosaturated fatty acids (MUFA) and polyunsaturated fatty acids (PUFA) in the lean meat was higher than that in the skin (shown in supplementary data, Table S1), which might be conductive to cholesterol oxidation. The result was in accordance with Chen et al.’s [17] study, who found that the total amount of COPs in pig feet meat was significantly higher than those in pig feet skin during a heating period of 0–4 h. They noted that the phospholipids in the meat membrane consisted of more polyunsaturated fatty acids, which could accelerate cholesterol oxidation during heating [29]. In addition, protein denaturation in the lean meat during heating might lead to the release of haem or non-haem ions that promote cholesterol oxidation [30]. Regarding the subcutaneous fat, cholesterol oxidation might be slow in the lipid matrix, which has a high degree of saturation (shown in supplement data, Table S1). Lipids may compete for oxygen with sterols and reduce sterol oxidation by auto-oxidation [31].

In addition, MeIQ, MeIQx, 7,8-DiMeIQx, 4,8-DiMeIQx, Trp-P-2, PhIP, Trp-P-1, and MeAαC were not detected in all the samples. This outcome is different from Lan and Chen’s [16] results, which showed that seven HAs, including IQ, MeIQx, MeIQ, 4,8-DiMeIQx, Trp-P-1, PhIP, and AαC, were formed in marinated pork cooked with 1% rock candy and 20% soy sauce. For reheating, IQ was not detected in all of the samples. For the cooking cycle times of marinating juice, IQ was detected after 4 cycles, and its content was the highest of four HAs. It was reported that IQ could be formed through the interactions between creatinine and amino acids during heating [32]. In addition, the reducing sugars were responsible for the synthesis of the pyridine part of IQ [33]. Pearson et al. [34] proposed that IQ was formed by the reaction of dialkyl radicals with creatinine. Kikugawa [35] confirmed the free radical mechanism for the formation of IQ. The amounts of free radicals and precursors in the marinating juice increased with the cooking cycle times, which could account for the formation of IQ after 4 cycles.

Only Norharman, Harman, and AαC could be detected in all samples, and the content of AαC was relatively low compared to content of the other HAs. The results were different from Lan and Chen’s [16] work, who reported that Harman and Norharman were not detected in marinated pork cooked with 1% rock candy and 20% soy sauce. However, another report showed that Harman and Norharman formed easily in braised chicken [18]. This outcome might be explained by the addition of different ingredients to the meat samples during marinating and the varying proportions of HAs precursors in different meats. Pan et al. [36] demonstrated that soy sauce might be the critical factor affecting the formation of Harman and Norharman during the marinating process. Their results showed that the amount of Harman and Norharman in five kinds of soy sauce were 111.47 to 301.30 ng/g and 80.76 to 199.27 ng/g. In mutton marinated with soy sauce, the amounts of Harman and Norharman were 17.30 and 12.18 ng/g, which were 7 and 10 times higher than those in mutton marinated without soy sauce. Therefore, Harman and Norharman in marinated meat could be derived from soy sauce.

The results clearly showed that the repeated use of the marinating juice might account for the increase in the HAs in the marinated hock. Yao et al. [18] found that 68.80 ng/g and 96.98 ng/g of total HAs were generated in braised chicken and the juice after 20 cycles; these amounts were 7 and 6.4 times, respectively, higher than those after the 5th cycle. In a similar study, Wang et al. [21] reported that the number of HAs in fried fish patties increased with the frying cycle. After 50 cycles, the total amount of HAs detected was 3 ng/g, which was 1.6 times higher than that detected after the 5th cycle.

In addition, the results revealed that reheating also promotes the formation of HAs. This was similar to Lan et al.’s [37] study, who found that the total level of HAs in marinated pork increased from 13.92 ng/g (heating at 98 ± 2 °C for 4 h) to 32.98 ng/g (heating at 98 ± 2 °C for 32 h). Reheating served as another heating process in addition to the marinating process and resulted in the substantial generation of HAs. Yao et al. [20] demonstrated that the total amount of HAs in commercial braised sauce beef that was sterilized ranged from 9.99 to 27.15 ng/g, whereas the unsterilized samples contained only 4.33–8.45 ng/g of total HAs. The marinated hock was prepared by boiling the hock with soy sauce and sugar, which contained an adequate amount of HA precursors, such as amino acids, reducing sugar and creatinine. The formation rate of reducing sugar or the reaction rate with amino acids or creatinine varied depending upon the length of the heating time [20]. These results explained the increase in HAs after reheating.

Notably, the total HAs generated in the skin were higher than those generated in the subcutaneous fat and lean meat after a given cycle (*p* < 0.05). Yao et al. [18] found that the amount of the total HAs in the skin and meat of chicken boiled for 6 h were 25.69 and 17.41 ng/g. The skin was exposed directly to the heat source and marinating juice, possibly explaining the higher HAs detected in the skin [38].

Interestingly, the amount of COPs and HAs was both increased with the increasing cooking cycle times and after reheating. During lipid peroxidation, alcohols, aldehydes, ketones, organic acids and even a number of compounds such as N-heterocyclics might be formed. In addition, free radicals are formed during lipid peroxidation, which is conductive to the formation of HAs [39,40]. In a word, the lipid peroxidation facilitates the formation of HAs.

In summary, the present study revealed that the cooked cycle times of the marinated juice and reheating promoted the formation of COPs and HAs in marinated pig hock. A relatively high amount of COPs and HAs were formed after cooking in the recycled marinated juice and reheating. Because marinated pig hock is widely consumed in China, the effects of marinated pig hock on the daily intake of COPs and HAs should not be ignored. Therefore, it is important to evaluate their health risk to humans and develop strategies to reduce intake of COPs and HAs. From the results of this paper, there are two possible ways to reduce the COPs and HAs content in marinated pig hock:

1. Use fresh marinated juice instead of recycled marinated juice.
2. Reduce the reheating time and temperature, or using non-thermal sterilization technology.

Essentially, novel technology and more strategies are needed to reduce the formation of COPs and HAs in marinated pig hock.

## 5. Conclusions

In conclusion, the formation of COPs accompanied the formation of HAs during marinated pig hock processing, and the cooked cycle times of the marinated juice and reheating promoted the formation of total COPs and HAs in marinated pig hock skin, subcutaneous fat, and lean meat. Both triol and IQ were detected after the 4th cycle. Reheating resulted in increases in the contents of COPs and HAs in the marinated hock, and no significant difference was found between different reheating temperatures. In addition, the content of COPs in the lean meat was the highest, while the content of HAs in the skin was the highest. The results indicated that one of the possible strategies for mitigating the formation of COPs and HAs during marinated pig hock processing was to adjust the process condition. However, further study should be performed to continue development of effective strategies to mitigate the formation of COPs and HAs, to maintain and even improve the nutritional quality of marinated pig hock as well as to maintain a good shelf-life. Moreover, the results of the present work would raise the public awareness of the safety of traditional Chinese food commodities, which was of great importance to meat manufacturers for reducing the formation and concentrations of COPs and HAs in meat processing, facilitating the upgrading of traditional food processing technology.

## Figures and Tables

**Table 1:** Effect of cooked cycle times of marinated juice on the formation of COPs in marinated pig hock skin, subcutaneous fat and lean meat.

COPs (ng/g)	Cycles ^x^			
	1	4	8	12
**Skin**				
7β-OH	165.0 ± 47.7 ^d^	306.4 ± 8.2 ^c^	391.9 ± 21.0 ^b^	700.7 ± 33.1 ^a^
5,6α-EP	ND ^y^	ND	ND	ND
Triol	ND	169.0 ± 1.1 ^a^	169.0 ± 1.4 ^a^	168.1 ± 0.3 ^a^
25-OH	154.7 ± 20.4 ^b^	177.3 ± 3.7 ^ab^	171.3 ± 8.6 ^ab^	194.4 ± 0.5 ^a^
7-keto	276.5 ± 34.6 ^d^	464.7 ± 20.9 ^c^	605.2 ± 56.6 ^b^	905.1 ± 93.6 ^a^
Total COPs	596.3 ± 62.7 ^cC^	1117.3 ± 26.4 ^bB^	1337.3 ± 67.5 ^bB^	1968.4 ± 103.3 ^aB^
**Subcutaneous fat**				
7β-OH	237.1 ± 28.4 ^b^	363.3 ± 43.3 ^a^	455.7 ± 51.7 ^a^	465.8 ± 61.2 ^a^
5,6α-EP	ND	ND	ND	ND
Triol	ND	168.3 ± 0.4 ^a^	168.4 ± 1.8 ^a^	167.2 ± 0.6 ^a^
25-OH	181.4 ± 11.0 ^b^	191.8 ± 6.7 ^b^	219.9 ± 2.5 ^a^	220.4 ± 6.4 ^a^
7-keto	380.3 ± 31.2 ^c^	530.7 ± 32.1 ^b^	727.5 ± 62.8 ^a^	716.6 ± 27.1 ^a^
Total COPs	798.8 ± 70.6 ^cB^	1254.1 ± 82.5 ^bB^	1571.5 ± 113.8 ^aAB^	1570.0 ± 95.4 ^aC^
**Lean meat**				
7β-OH	482.8 ± 30.4 ^c^	583.0 ± 46.4 ^b^	650.2 ± 76.1 ^b^	935.7 ± 48.2 ^a^
5,6α-EP	ND	ND	ND	ND
Triol	ND	178.7 ± 11.1 ^a^	174.3 ± 8.1 ^a^	197.8 ± 19.4 ^a^
25-OH	152.8 ± 4.3 ^b^	154.6 ± 2.5 ^b^	158.3 ± 1.7 ^b^	174.4 ± 5.7 ^a^
7-keto	569.6 ± 46.7 ^c^	782.2 ± 93.5 ^b^	790.7 ± 109.8 ^b^	1027.8 ± 120.1 ^a^
Total COPs	1205.2 ± 92.0 ^cA^	1698.5 ± 145.4 ^bA^	1773.4 ± 176.2 ^bA^	2335.7 ± 125.1 ^aA^

^x^ Values were expressed as the means ± standard deviation, and values with different superscript letters in the same row were significantly different. For total COPs only: A-C denote values with different letters in the same column were significantly different). ^y^ ND = not detected.

**Table 2:** Effect of the cooked cycle times of the marinated juice on the formation of the HAs in the marinated pig hock skin, subcutaneous fat and lean meat.

HAs (ng/g)	Cycles ^x^			
	1	4	8	12
**Skin**				
IQ	ND ^y^	13.26 ± 0.64 ^b^	22.25 ± 1.72 ^a^	22.46 ± 1.80 ^a^
Norharman	3.97 ± 0.29 ^c^	7.68 ± 0.26 ^b^	13.13 ± 0.63 ^a^	14.33 ± 1.31 ^a^
Harman	4.80 ± 0.38 ^c^	8.01 ± 0.22 ^b^	15.94 ± 0.84 ^a^	15.95 ± 1.34 ^a^
AαC	0.34 ± 0.03 ^b^	0.39 ± 0.01 ^a^	0.41 ± 0.01 ^a^	0.42 ± 0.01 ^a^
Total HAs	9.11 ± 0.40 ^cA^	29.34 ± 0.60 ^bA^	51.73 ± 2.44 ^aA^	53.16 ± 2.56 ^aA^
**Subcutaneous fat**				
IQ	ND	10.60 ± 0.90 ^b^	14.16 ± 1.68 ^a^	15.79 ± 1.32 ^a^
Norharman	2.99 ± 0.25 ^c^	5.81 ± 0.70 ^b^	10.91 ± 1.09 ^a^	11.61 ± 1.44 ^a^
Harman	2.77 ± 0.21 ^c^	4.54 ± 0.65 ^b^	8.45 ± 0.83 ^a^	9.35 ± 1.13 ^a^
AαC	0.45 ± 0.03 ^a^	0.46 ± 0.04 ^a^	0.44 ± 0.02 ^a^	0.48 ± 0.01 ^a^
Total HAs	6.21 ± 0.29 ^cB^	21.41 ± 1.29 ^bB^	33.96 ± 1.81 ^aB^	37.23 ± 2.02 ^aB^
**Lean meat**				
IQ	ND	9.07 ± 0.12 ^b^	13.34 ± 1.42 ^a^	13.07 ± 0.99 ^a^
Norharman	2.46 ± 0.16 ^c^	4.16 ± 0.13 ^b^	8.06 ± 0.61 ^a^	8.86 ± 0.17 ^a^
Harman	2.85 ± 0.73 ^c^	5.34 ± 0.03 ^b^	9.82 ± 0.61 ^a^	9.78 ± 0.11 ^a^
AαC	0.41 ± 0.00 ^a^	0.43 ± 0.01 ^a^	0.43 ± 0.02 ^a^	0.44 ± 0.02 ^a^
Total HAs	5.71 ± 0.89 ^cB^	19.00 ± 0.13 ^bC^	31.64 ± 1.17 ^aB^	32.15 ± 1.02 ^aC^

^x^ Values were expressed as the means ± standard deviation, and the values with different superscript letters in the same row were significantly different. For total HAs only: A-C denote values with different letters in the same column and were significantly different. ^y^ ND = not detected.

**Table 3:** Effects of reheating on the formation of COPs in the marinated pig hock skin, subcutaneous fat, and lean meat.

COPs (ng/g)	Methods ^x^		
	Unreheated	95 °C, 30 min	121 °C, 25 min
**Skin**			
7β-OH	198.8 ± 6.0 ^b^	241.8 ± 15.7 ^a^	233.1 ± 13.2 ^a^
5,6α-EP	ND ^y^	ND	ND
Triol	ND	ND	ND
25-OH	151.5 ± 1.6 ^b^	166.9 ± 6.7 ^a^	175.6 ± 3.8 ^a^
7-keto	308.2 ± 3.8 ^c^	426.0 ± 25.8 ^b^	411.7 ± 10.0 ^a^
Total COPs	658.5 ± 3.8 ^bC^	834.7 ± 34.8 ^aB^	820.4 ± 12.4 ^aC^
**Subcutaneous fat**			
7β-OH	238.4 ± 11.9 ^b^	297.0 ± 12.4 ^a^	300.4 ± 24.0 ^a^
5,6α-EP	ND	ND	ND
Triol	ND	ND	ND
25-OH	163.8 ± 1.0 ^b^	177.2 ± 7.8 ^a^	171.3 ± 3.0 ^a^
7-keto	393.0 ± 10.7 ^b^	400.5 ± 8.5 ^b^	435.5 ± 13.3 ^a^
Total COPs	795.1 ± 2.2 ^bB^	874.8 ± 28.7 ^aB^	907.2 ± 26.3 ^aB^
**Lean meat**			
7β-OH	499.8 ± 44.2 ^b^	779.3 ± 86.0 ^a^	948.3 ± 143.7 ^a^
5,6α-EP	ND	ND	ND
Triol	ND	ND	ND
25-OH	144.3 ± 1.8 ^a^	150.0 ± 0.4 ^a^	148.1 ± 2.8 ^a^
7-keto	526.5 ± 23.4 ^b^	621.6 ± 26.0 ^a^	642.3 ± 28.5 ^a^
Total COPs	1170.6 ± 59.4 ^bA^	1550.9 ± 94.4 ^aA^	1738.7 ± 165.0 ^aA^

^x^ Values were expressed as the means ± standard deviation, and the values with different superscript letters in the same row were significantly different (*p* < 0.05). For total COPs only: A-C denote values with different letters in the same column and were significantly different (*p* < 0.05). ^y^ ND = not detected.

**Table 4:** Effect of reheating on the formation of heterocyclic amines (HAs) in the marinated pig hock skin, subcutaneous fat, and lean meat.

HAs (ng/g)	Methods ^x^		
	unreheated	95 °C, 30 min	121 °C, 25 min
**Skin**			
Norharman	3.48 ± 0.16 ^c^	4.14 ± 0.18 ^b^	5.44 ± 0.15 ^a^
Harman	4.49 ± 0.34 ^c^	6.90 ± 0.61 ^b^	9.04 ± 0.11 ^a^
AαC	0.16 ± 0.01 ^c^	0.32 ± 0.01 ^b^	0.36 ± 0.01 ^a^
Total HAs	8.13 ± 0.79 ^cA^	11.36 ± 0.77 ^bA^	14.84 ± 0.26 ^aA^
**Subcutaneous fat**			
Norharman	2.91 ± 0.15 ^b^	3.46 ± 0.14 ^a^	3.74 ± 0.58 ^a^
Harman	2.87 ± 0.10 ^b^	3.33 ± 0.41 ^ab^	3.60 ± 0.41 ^a^
AαC	0.25 ± 0.05 ^b^	0.41 ± 0.02 ^a^	0.42 ± 0.02 ^a^
Total HAs	6.03 ± 0.10 ^bB^	7.20 ± 0.54 ^aB^	7.76 ± 1.00 ^aB^
**Lean meat**			
Norharman	2.46 ± 0.39 ^a^	2.61 ± 0.33 ^a^	3.03 ± 0.70 ^a^
Harman	2.39 ± 0.21 ^a^	2.72 ± 0.13 ^ab^	3.41 ± 0.54 ^a^
AαC	0.23 ± 0.01 ^c^	0.30 ± 0.01 ^b^	0.43 ± 0.02 ^a^
Total HAs	5.08 ± 0.31 ^bC^	5.63 ± 0.27 ^abC^	6.87 ± 0.91 ^aB^

^x^ Values were expressed as the means ± standard deviation, and the values with different superscript letters in the same row were significantly different. For total HAs only: A-C denote values with different letters in the same column and were significantly different.

## Supplementary Materials

The following are available online at https://www.mdpi.com/2304-8158/9/8/1104/s1, Table S1: Acid profile of pig hock skin, subcutaneous fat and lean meat (mg/g) (*n* = 3).
