# Supplementary material for: Effects of Cooking Cycle Times of Marinating Juice and Reheating on the Formation of Cholesterol Oxidation Products and Heterocyclic Amines in Marinated Pig Hock
Source: Foods. 2020 Aug 12;9(8):1104. doi: 10.3390/foods9081104 (PMC7466265; doi:10.3390/foods9081104)
Supplement: Supplementary file 1 [file foods-09-01104-s001.pdf]

1. Method

1.1 Analysis of free fatty acid composition

Total lipids were extracted from 5 g of raw pig kock skin, subcutaneous fat and lean meat according to the method described by Folch et al. [1] using 25 ml of chloroform: methanol (2 : 1) as the solvent.

The dry extracted lipids was separated using NH2-aminopropyl minicolumns, following the method of Kaluzny et al. [2].

Free fatty acids (FFAs) were eluted with 3.0 ml of diethyl ether: acetic acid 2%. The amounts of FFAs was expressed as mg/g total lipids.Fatty acids composition was analyzed using gas–liquid chromatography. Gas chromatograph (Trace GC Ultra, Thermo Electron Corporation, Waltham, USA) was used to analyze the methyl esters of fatty acids as reported by Morrison and Smith [3].

2. Result

Table 1 Acid profile of pig hock skin, subcutaneous fat and lean meat (mg/g) (n=3)

FFA	skin	subcutaneous fat	lean meat
C14:0	1.46 ±0.04b	12.15±1.19a	2.04±0.08b
C16:0	21.37±0.82b	211.25±15.33a	34.26±2.36b
C18:0	3.77± 0.24b	110.21±8.74a	13±0.97b
SFA	26.6±0.63c	333.62±16.61a	49.30±3.40b
C16:1	14.14±0.46 b	18.05±1.00a	5.47±0.40c
C18:1	29.01±0.57c	367.85±12.84a	55.44±4.79b
C20:1	0.11± 0.02b	3.06±0.36a	0.25±0.04a
MUFA	43.26±0.99c	388.96±12.17a	61.16±5.23b
C18:2	21.75±2.20 b	97.69±6.15a	37.33±1.12b
C18:3	1.75±0.14b	21.81±1.90a	1.05±0.07b
PUFA	23.50±2.08c	119.50±8.05a	38.38±1.05b

Different lower-case letters in the same row indicate signicant differences during the processing ($P < 0.05$) .

SFA, saturated fatty acids; MUFA, monosaturated fatty acids; PUFA, polyunsaturated fatty acid .

References

[1] Folch, J., Lees, M., & Sloane Stanley, G. H. A simple method for the isolation and purification of total lipides from animal tissues. *J. Biol. Chem.* **1957**, 226, 497-509.

[2] Kaluzny, M. A., Duncan, L. A., Merritt, M. V. & Epps, D.E. Rapid Separation of Lipid Classes in High Yield and Purity Using Bonded Phase Columns. *J. Lipid. Res.* **1985**, 26, 135-140.

[3] Morrison, W. R, & Smith, L. M. Preparation of fatty acid methyl esters and dimethylacetals from lipids with boron fluoride--methanol. *J. Lipid. Res*. **1964**, 5, 600-608.
